# NatB-mediated protein N-α-terminal acetylation is a potential therapeutic target in hepatocellular carcinoma

**DOI:** 10.18632/oncotarget.17332

**Published:** 2017-04-21

**Authors:** Leire Neri, Marta Lasa, Alberto Elosegui-Artola, Delia D'Avola, Beatriz Carte, Cristina Gazquez, Sara Alve, Pere Roca-Cusachs, Mercedes Iñarrairaegui, Jose Herrero, Jesús Prieto, Bruno Sangro, Rafael Aldabe

**Affiliations:** ^1^ Gene Therapy and Regulation of Gene Expression Program, Centro de Investigación Médica Aplicada, Universidad de Navarra, Pamplona, Spain; ^2^ Institute for Bioengineering of Catalonia, Barcelona, Spain; ^3^ Liver Unit, Clínica Universidad de Navarra, Centro de Investigación Biomédica en Red en el Área Temática de Enfermedades Hepáticas y Digestivas (Ciberehd), Pamplona, Spain; ^4^ Instituto de Investigación Sanitaria de Navarra (IdiSNA), Pamplona, Spain; ^5^ Department of Biology, CBMA-Centre of Molecular and Environmental Biology, University of Minho, Campus de Gualtar, Braga, Portugal; ^6^ University of Barcelona, Barcelona, Spain

**Keywords:** tropomyosin, CDK2, focal adhesions, cell-cell junctions, cell cycle arrest

## Abstract

The identification of new targets for systemic therapy of hepatocellular carcinoma (HCC) is an urgent medical need. Recently, we showed that hNatB catalyzes the N-α-terminal acetylation of 15% of the human proteome and that this action is necessary for proper actin cytoskeleton structure and function. In tumors, cytoskeletal changes influence motility, invasion, survival, cell growth and tumor progression, making the cytoskeleton a very attractive antitumor target. Here, we show that hNatB subunits are upregulated in in over 59% HCC tumors compared to non-tumor tissue and that this upregulation is associated with microscopic vascular invasion. We found that hNatB silencing blocks proliferation and tumor formation in HCC cell lines in association with hampered DNA synthesis and impaired progression through the S and the G2/M phases. Growth inhibition is mediated by the degradation of two hNatB substrates, tropomyosin and CDK2, which occurs when these proteins lack N-α-terminal acetylation. In addition, hNatB inhibition disrupts the actin cytoskeleton, focal adhesions and tight/adherens junctions, abrogating two proliferative signaling pathways, Hippo/YAP and ERK1/2. Therefore, inhibition of NatB activity represents an interesting new approach to treating HCC by blocking cell proliferation and disrupting actin cytoskeleton function.

## INTRODUCTION

Acetylation is a post-translational modification of cellular proteins consisting of covalent binding of an acetyl group from an acetyl-CoA donor. This reaction is catalyzed by two families of acetyltransferases: lysine acetyltransferases (KATs) [1] and alpha-aminoterminal acetyltransferases (NATs) [2]. The NAT family has 6 enzymatic complexes, the majority of which are formed by a catalytic and an auxiliary subunit. NATs acetylate co-translational nascent polypeptides and each complex targets distinct amino-terminal protein sequences selected based on the first two aminoacids [2]. The hNatB enzymatic complex, which is composed of an NAA20 catalytic moiety and an NAA25 accessory subunit, acetylates all proteins with a glutamine, asparagine, glutamic acid or aspartic acid residue immediately after the initial methionine [3].

It has been shown that the expression of NAT subunits is deregulated in tumor samples and that NAT complexes are required for the proliferation and survival of some cancer cell lines [4]. Upregulation of the NatA catalytic subunit, NAA10, has been reported in lung, breast and urinary bladder cancers as well as in cervical carcinoma and HCC in association with poor survival and tumor aggressiveness [5]. Additionally, the NatA accessory subunit NAA15 was found to be upregulated in rapidly progressing neuroblastomas and in undifferentiated papillary thyroid carcinomas [6, 7]. Conversely, NAA10 downregulation may favor the progression of other tumors and its upregulation was reported to be associated with better prognosis, smaller tumors and reduced metastasis [8]. Moreover, NatD has been found to be significantly downregulated in hepatocellular carcinoma samples and is implicated in apoptosis regulation [9, 10], while the NatC catalytic subunit NAA30 is highly expressed in glioblastoma patients [11]. Our group has previously shown that in HCC, the expression of the NatB catalytic subunit NAA20 correlates with tumor progression [12]. In addition several authors have observed a direct correlation between NatB subunits expression and cellular proliferation [13, 14].

In cancer cells cytoskeletal function is essential for cell division, cell migration and cellular transformation, thus representing an attractive target for antitumor therapy. Because inhibitors of actin microfilament polymerization are highly toxic due to their inability to discern between the tumor actin cytoskeleton and sarcomeric actin filaments, attention has been focused on actin regulatory proteins, as they are differentially expressed in tumor and normal cells [15]. Tropomyosins are actin cytoskeleton regulatory proteins possessing high number of isoforms resulting from either different gene products or alternative splicing [16]. Tropomyosin forms head-to-tail homo- or heterodimers along actin filaments regulating their biophysical properties and interactions with other actin binding proteins in an isoform specific manner [16]. We have previously reported that NatB activity is crucial for the maintenance of actin filaments structure and function by acetylating tropomyosin at its amino terminus, a modification in some long tropomyosin isoforms that is required for interacting with actin-binding proteins or modulate actin-binding and polymerization [3, 17]. In contrast, it has been shown that the actin cytoskeleton is required for cell cycle progression and that disruption of actin filaments causes G1 arrest and impaired cytokinesis, which is in agreement with its role in spindle formation and contractile ring formation. In addition, actin is also implicated in cell signaling pathways, including Hippo, Ras-MAPK, PI3K and NF-kB, that are critical in the control of cell growth, cell size and cell motility.

In the present study, we analyzed the potential role of NatB-mediated protein alpha-aminoterminal-acetylation in the growth and progression of HCC. We observed that hNatB is upregulated in large number of human hepatocellular carcinomas in correlation with microvascular invasion. We found that hNatB is required for N-α-terminal acetylation of two tropomyosin isoforms, tropomyosin 1.6 (TPM 1.6) and tropomyosin 2.1 (TPM 2.1) and that inhibition of hNatB causes the disruption of focal and intercellular adhesions, blocking cell cycle progression in association with degradation of CDK2, a cyclin-dependent kinase that is also a hNatB substrate. Our data suggest that NatB inhibition may be a novel and promising approach for the treatment of HCC.

## RESULTS

### NatB subunits are upregulated in human hepatocellular carcinoma

**Figure 1:**
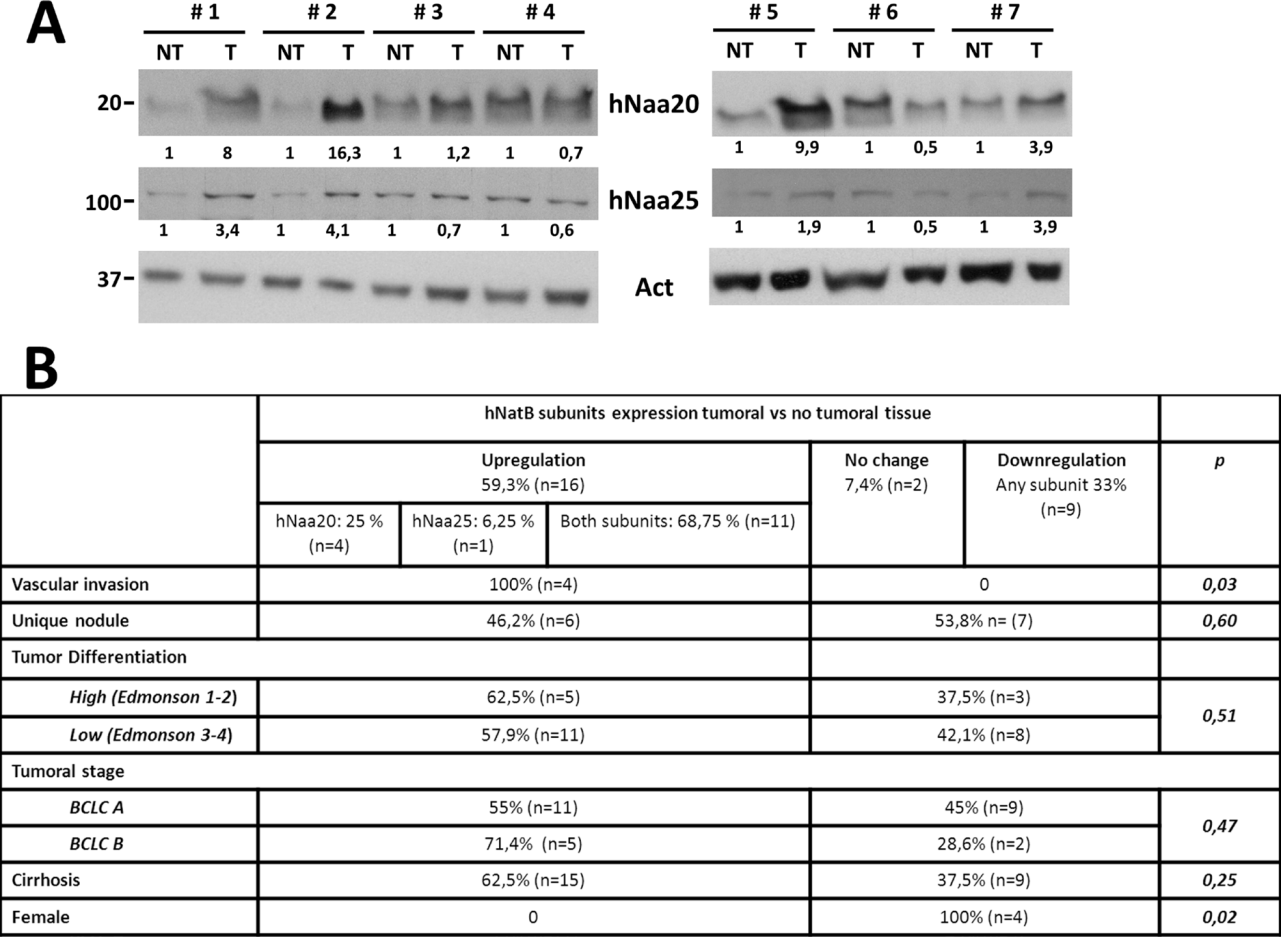
hNatB protein expression in human hepatocellular carcinoma (HCC) and non-tumor liver tissue (**A**) Western blot of hNatB subunits, NAA20 and NAA25, in the non-tumor (NT) and tumor (T) liver tissue of patients with HCC. Images were quantified, normalized with obtained actin values and compared NT vs T paired samples, being considered NT sample as 1. (**B**) Relationship between NAA20 and NAA25 expression and HCC characteristics.

In a previous work we found an increased expression of the NatB catalytic subunit, NAA20, in mouse and human HCC [12]. To better define the role of hNatB in human HCC development we have now analyzed both NAA20 and NAA25 protein expression in paired tumor and non-tumor samples from 27 HCC patients (74% stage BCLC A and 26% stage B) treated with liver resection or transplantation (Figure 1, Supplementary Figure 1). In 59% of patients hNatB subunits were upregulated in tumor tissue compared to non-tumor tissue while 33% presented reduced tumor expression of both NatB components. Most samples manifesting hNatB upregulation showed increased expression of both NatB subunits (11 of 16 samples) and only 4 samples exhibited enhanced levels of the catalytic moiety in isolation. To our knowledge there are no previous data regarding the upregulation of the complete NAT complex in connection to development of human tumors. Interestingly we observed a significant correlation between upregulation of the two NatB subunits and microscopic vascular invasion (*p* = 0.04) indicating that increased levels of the hNatB enzymatic complex in HCC can predict poor prognosis. No association was found between NatB expression and tumor stage, multinodularity or tumor cell differentiation.

### NatB activity controls the structure and function of the actin cytoskeleton in HCC

**Figure 2:**
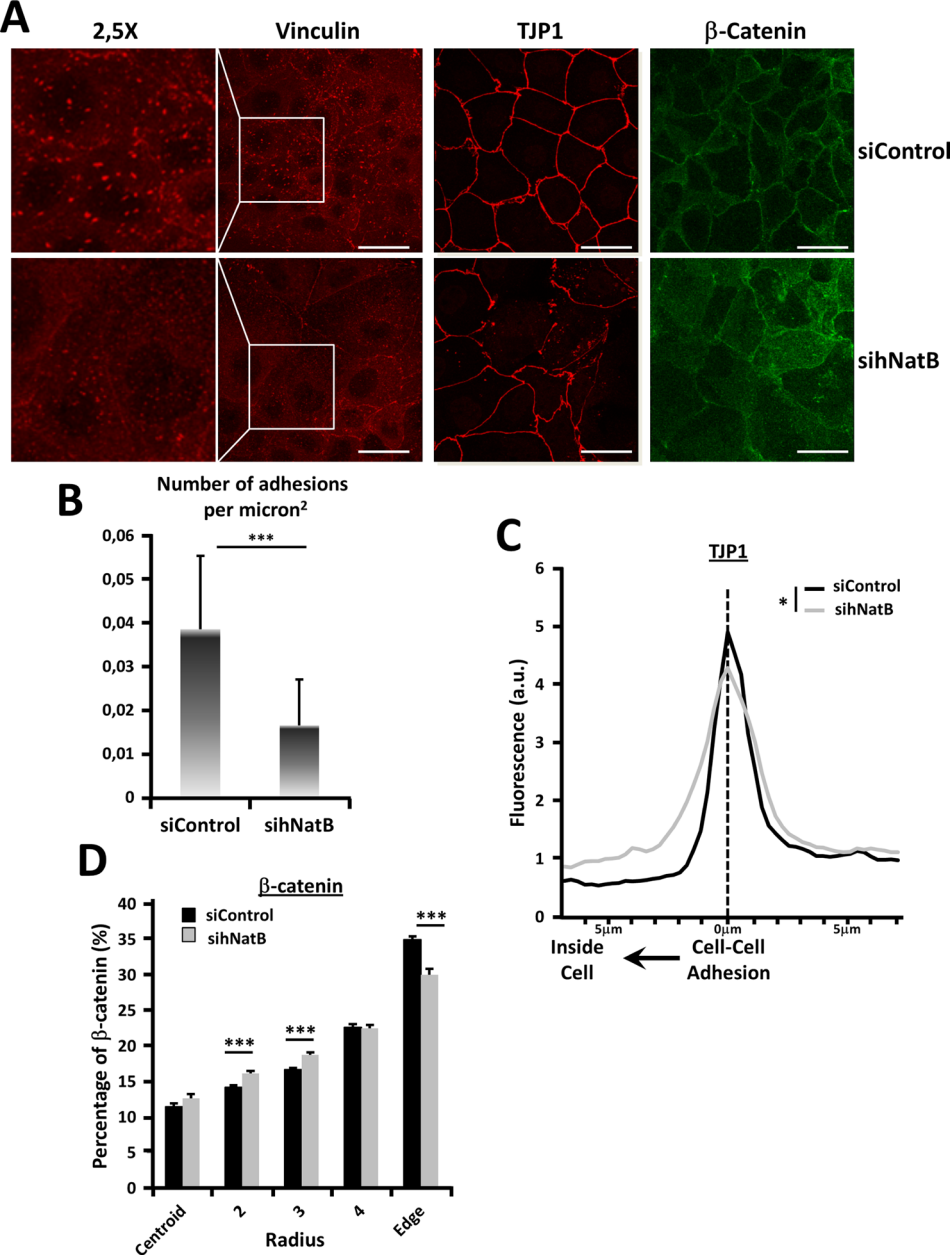
Effects of hNatB downregulation on focal adhesions and cell-cell interactions on PLC/PRF/5 cells PLC/PRF/5 cells were transfected with negative control siRNA (siControl) or hNaa20 and hNaa25 siRNAs (sihNatB) and 96 hours later were paraformaldehyde fixed and analyzed with specific antibodies for immunofluorescence analysis (**A**) with specific antibodies for focal adhesions (vinculin) and cell-cell contacts: tight junctions (TJP1) and adherens junctions (β-catenin). (**B**) Focal adhesions were analyzed and the number of adhesions per micron^2^ were quantified. (**C**) TJP1 presence was quantified from the cell-cell contact point to 7mm inside the cell (*n* = 10). (**D**) For β-catenin distribution quantification cells were transformed into circular surfaces, divided in 5 concentric circular rings with an equal surface ranging from the cell center (centroid) to the cell membrane (Edge) and % of β-catenin present in each region was quantified (*n* > 21). The data are presented as the mean ± SME. Statistical significance is shown in figure panels and it was set at *p* < 0.05 (*), *p* < 0.01 (**) and *p* < 0.001 (***) value. Unpaired (B, D) and paired *t-test* (C) were performed. Scale bar: 25 μm.

**Figure 3:**
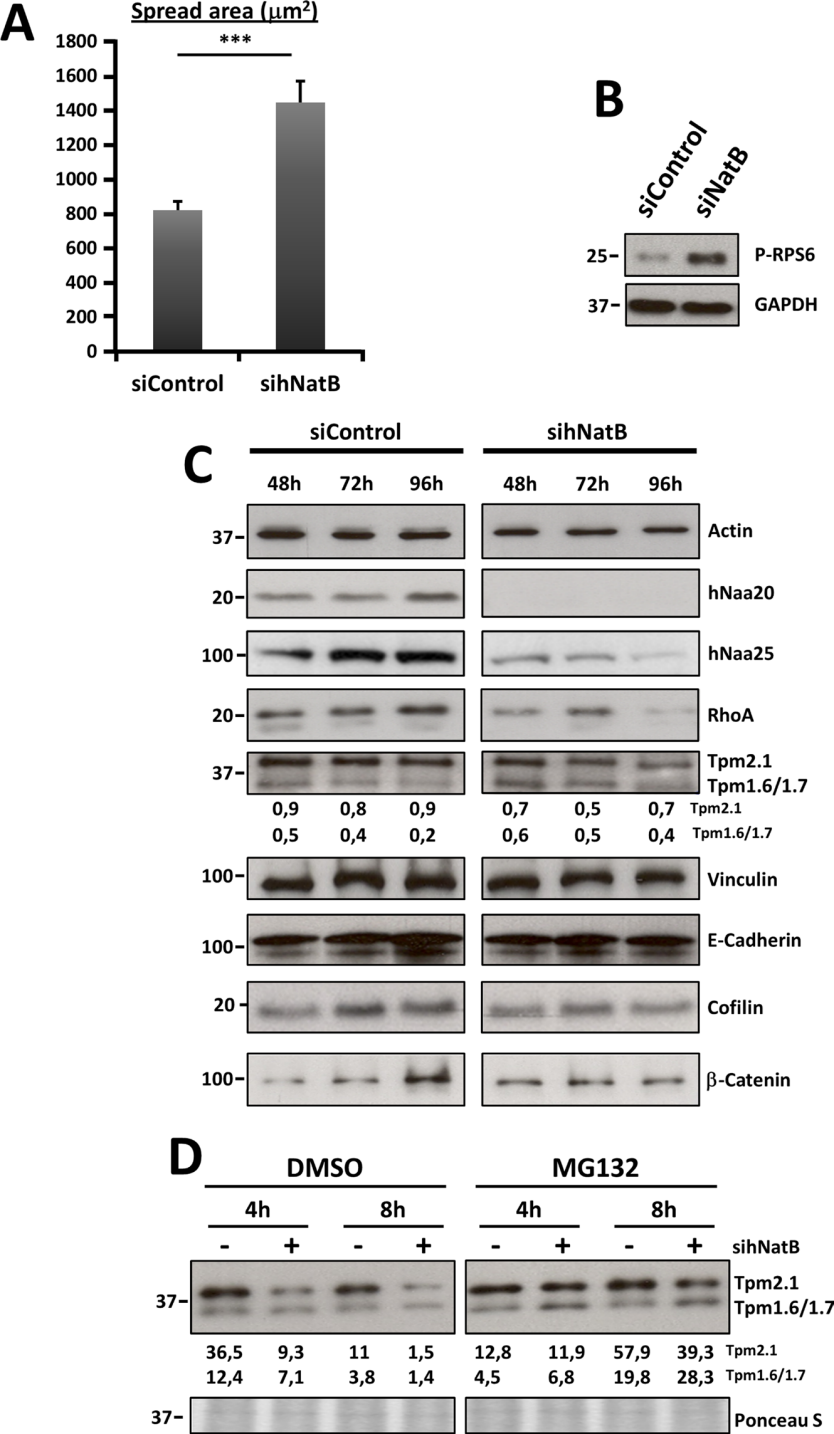
Effects of hNatB downregulation on cell size and actin cytoskeleton protein expression on PLC/PRF/5 cells (**A**) Cell spread area corresponds to the average cell surface in mm^2^ in both experimental conditions (*n* > 29). (**B**) mTOR activation was evaluated 96 hours after siRNAs transfection studying Ribosomal S6 protein (P-RPS6) by western blot in cell lysates. (**C**) PLC/PRF/5 cells transfected with negative control siRNA (siControl) or *NAA20* and *NAA25* siRNAs (sihNatB) lysates were harvested at different time points after transfection and western blot analysis was performed using antibodies against RHOA, tropomyosins, Vinculin, E-Cadherin, cofilin and β-catenin. Actin was used for normalizing extracts and NAA20 and NAA25 to verify NatB downregulation. (**D**) Tropomyosin expression was evaluated by western blot in PLC/PRF/5 cells 96 hours after siNatB (+) and siControl (−) transfection. Cells were treated with proteasome inhibitor MG132 (1 mm) or DMSO for 4 or 8 hours and then cell lysates were prepared. The data are presented as the mean ± SME. Statistical significance is shown in figure panels and it was set at *p* < 0.05 (*), *p* < 0.01 (**) and *p* < 0.001 (***) value. Paired *t-test* was performed.

PLC/PRF/5 cells transfected with a negative control siRNA (siControl) or *NAA20* and *NAA25* siRNAs (sihNatB) were analyzed 96 hours later with specific antibodies for immunofluorescence (Figure 2A, Supplementary Figure 2) and western blot analysis. We found that hNatB knockdown, which reduces NatB-targeted N-terminal acetylation [3], caused a marked decrease in focal adhesions (Figure 2A, 2B) without affecting the amount of vinculin present in the cells, indicating redistribution of this focal adhesion protein (Figure 3C). These changes were associated with downregulation of RHOA (Figure 3C), a protein that regulates the formation of actin stress fibers and focal adhesion complexes [18].

We observed that TPM 1.6/1.7 and TPM 2.1 expression was affected upon NatB silencing in PLC/PRF/5 cells, with some variations among experiments (Figure 3C, 3D). This manoeuvre induced a decrease in TPM 2.1 protein without modifying its gene expression and an increase in *TPM 1.6/1.7* gene expression with a slight reduction in protein abundance (Figure 3C, 3D, Supplementary Figure 3A). MG-132 mediated proteasome inhibition resulted in restoration of TPM 2.1 to normal levels and an increased abundance of TPM 1.6/1.7, indicating that the amino-terminal acetylation of these proteins is needed for their stability (Figure 3D).

Epithelial cells are connected through intercellular tight junctions and adherens junctions that require an intact actin and cytoskeletal system for proper structure and function [19]. Adherens junctions transmit the tensile forces from outside of the cell to the actin cytoskeleton, while tight junctions separate the apical and the basolateral cell surface domains to maintain cell polarity. We therefore studied whether hNatB inhibition affects the integrity of adherens and tight junctions. We found that hNatB silencing caused a reduction in the TJP1 protein at the cell-cell interaction points (Figure 2C), which disappeared in some stretches (Figure 2A), revealing profound disruption of tight junctions. Moreover, occludin, another tight junction component, suffered similar distribution upon hNatB knockdown (Supplementary Figure 2). To characterize the fate of adherens junctions when hNatB was silenced, we analyzed the location of β-catenin in PLC/PRF/5 cells (Figure 2C). We observed that in control cells, β-catenin is predominantly located at the cellular edge associated with intercellular regions while in hNatB-deficient cells, it is partially relocated to the cytoplasm, revealing adherens junctions disorganization (Figure 2D). Interestingly, the accumulation of β-catenin in the cytoplasm is not accompanied by increased β-catenin transcriptional activity, as the expression of its target genes *CCND1* and *AXIN2* (Supplementary Figure 3C), is unmodified.

Quantification of the cell area revealed that hNatB knockdown in PLC/PRF/5 cells significantly increased the cell surface to almost twice its baseline (Figures 2A, 3A). One of the main signaling pathways that regulates cell size is the mTOR pathway [20]. Ribosomal Protein S6 (RPS6) was phosphorylated upon hNatB silencing indicating that deficient hNatB function promotes mTOR activation (Figure 3B).

### hNatB knockdown promotes CDK2 degradation and blocks cellular proliferation and tumor growth

**Figure 4:**
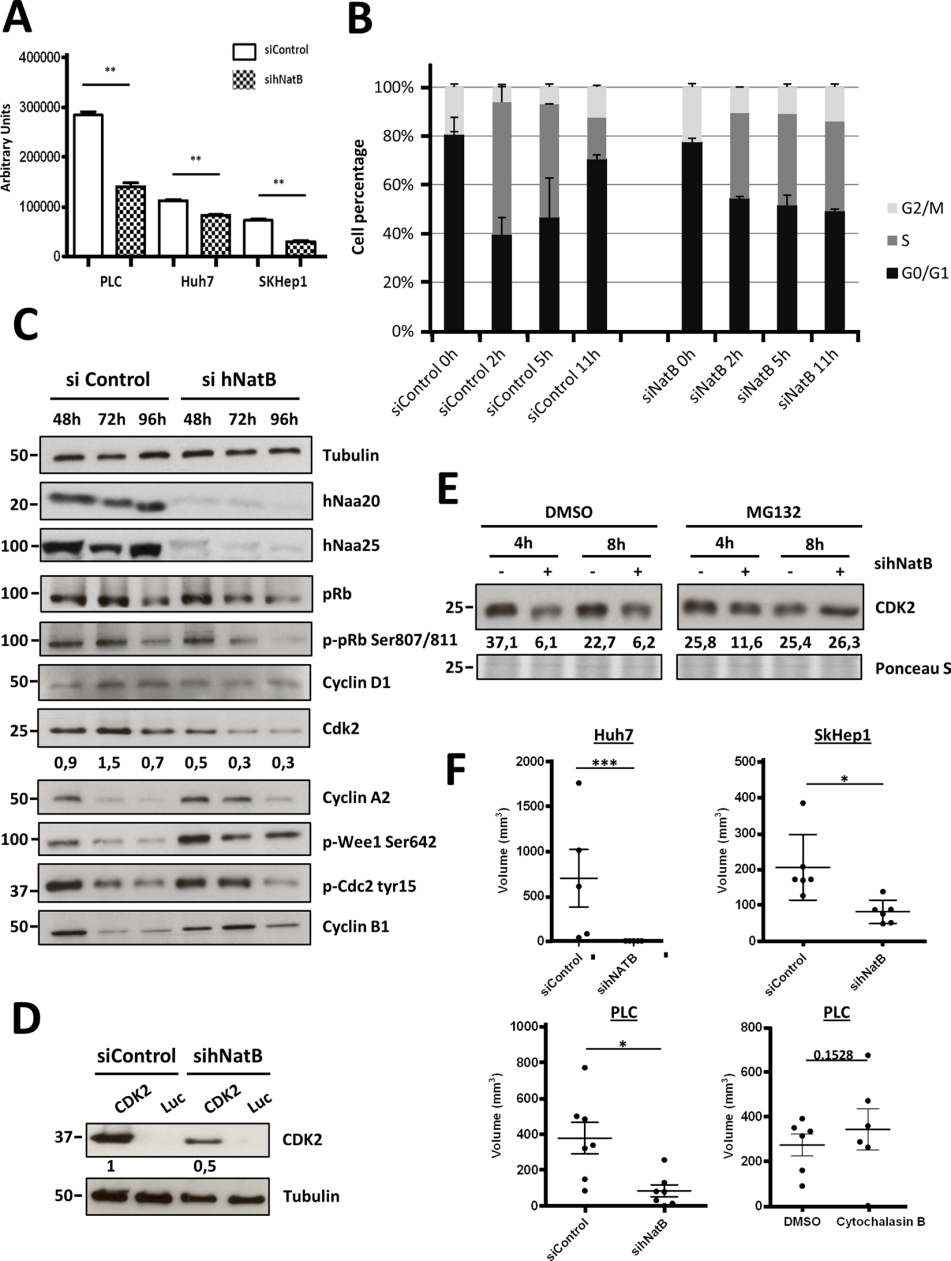
NatB is required for proper cell cycle progression and tumor growth PLC/PRF/5 cells were transfected with negative control siRNA (siControl) or hNaa20 and hNaa25 siRNAs (sihNatB) and 96 hours later proliferation rate was determined based on Cell Titer-Glo (**A**). siRNA transfected PLC/PRF/5 cells were synchronized in the early S phase with doble thymidine treatment, wash and analyze them by FACS 0, 2, 5 and 11 hours later. Before cells collection, they were incubated for 1 hour with Edu for DNA synthesis quantification (**B**). PLC/PRF/5 cell extracts were analyzed by western blot 48, 72 and 96 hours after transfection of control (siControl) and *NAA20* and *NAA25* (sihNatB) siRNAs visualizing diverse proteins that regulate cell cycle progression (**C**). In addition cells treated with control siRNAs or si*NAA20* and si*NAA25* (sihNatB) were transfected with *CDK2* or luciferase (Luc) expression vectors and overexpressed CDK2 was analyzed by western blot (**D**). CDK2 expression was evaluated by western blot in PLC/PRF/5 cells 96 hours after siNatB (+) and siControl (−) transfection. Cells were treated with proteasome inhibitor MG132 (1 mm) or DMSO for 4 or 8 hours and then cell lysates were prepared (**E**). Half million cells were subcutaneously injected into the flank of immunodeficient mice 48 hours after siRNA transfection or Cytochalasin B treatment and tumor volume was determined 60 days after cell inoculation (**F**). The data are presented as the mean ± SME of three experiments. Statistical significance is shown in figure panels and it was set at *p* < 0.05 (*), *p* < 0.01 (**) and *p* < 0.001 (***) value. Unpaired *t*-tests were performed.

In three different HCC cell lines hNatB silencing abated cellular proliferation (Figure 4A Supplementary Figure 6A), as has been observed in yeast, neurons and other human tumor cell lines [13, 14] and blocked DNA synthesis, as estimated by reduced EdU incorporation (Supplementary Figure 4). In cells synchronized at the G1/S phase transition with double thymidine treatment, hNatB inhibition hampered progression to the S phase, maintaining the percentage of cells in each phase of the cell cycle, while control cells exhibited dynamic cell cycle progression (Figure 4B). The disturbance of the cell cycle in hNatB-deficient cells was also revealed by observing impairement of DNA synthesis and alteration of dynamic Cyclin A2 and Cyclin B1 expression and CDC2 phosphorylation at different time points after releasing DNA synthesis blockade (Supplementary Figure 5).

To gain insight into the molecular mechanisms interfering with cell proliferation upon hNatB inhibition, we analyzed several cell cycle regulators at different time points after transfecting PLC/PRF/5 cells with anti-hNatB and control siRNAs (Figure 4C). We found that pRb was similarly expressed in control and hNatB-deficient cells while protein levels of CDK2, a key regulator of the S to G2/M phase transition, were markedly decreased after hNatB suppression. Interestingly, CDK2 mRNA levels remained unchanged (Supplementary Figure 3B) suggesting that the CDK2 protein was unstable in the absence of functional hNatB. CDK2 is a hNatB substrate that depends on this enzyme for its N-terminal acetylation [3]. To evaluate CDK2 stability in hNatB-deficient cells we overexpressed CDK2 in PLC/PRF/5 cells that had been treated with either anti-hNatB siRNA or control siRNA and found a marked decrease in CDK2 protein levels (Figure 4D). Interestingly, we also observed that reduced levels of CDK2 after hNatB inhibition in PLC/PRF/5 cells can be reversed by treatment with a proteasome inhibitor (Figure 4E), indicating that N-terminal acetylation is critical for CDK2 stability. Several groups have independently demonstrated that CDK2 inhibition causes a delay of the G2 phase and deregulation of CDC2/Cyclin B1 activity [21, 22]. Accordingly, we observed that CDK2 reduction is associated with upregulation of Cyclin A2 and Cyclin B1 and increased phosphorylation of WEE1 (Ser-642) and CDC2 (Tyr-15) 72 hours after siRNA transfection (Figure 4C). These data are consistent with restriction of late S phase and G2-M phase transition, in agreement with our observations in synchronized cells using cell cycle FACS analysis.

Since the above findings pointed to hNatB as a potential therapeutic target for HCC, we analyzed whether hNatB downregulation could reduce the tumorigenicity of human hepatocellular carcinoma cell lines subcutaneously inoculated in xenograft mouse models (Figure 4F). We found that the suppression of hNatB expression completely abolished the ability of Huh7 cells to form tumors and markedly reduced the volume of PLC/PRF/5 and Sk-Hep1 tumors seven weeks after cell transplantation. Similarly, when we treated PLC/PRF/5 cells with an actin depolymerizing agent, such as Cytochalasin B, which blocks cell proliferation and has antitumor activity [23], transplanted cells formed subcutaneous tumors as efficiently as control cells. Therefore, abrogation of hNatB activity could present a more pronounced and stable antitumor effect than Cytochalasin B.

### Disruption of ERK1/2 and Hippo signaling pathways in hNatB depleted cells

**Figure 5:**
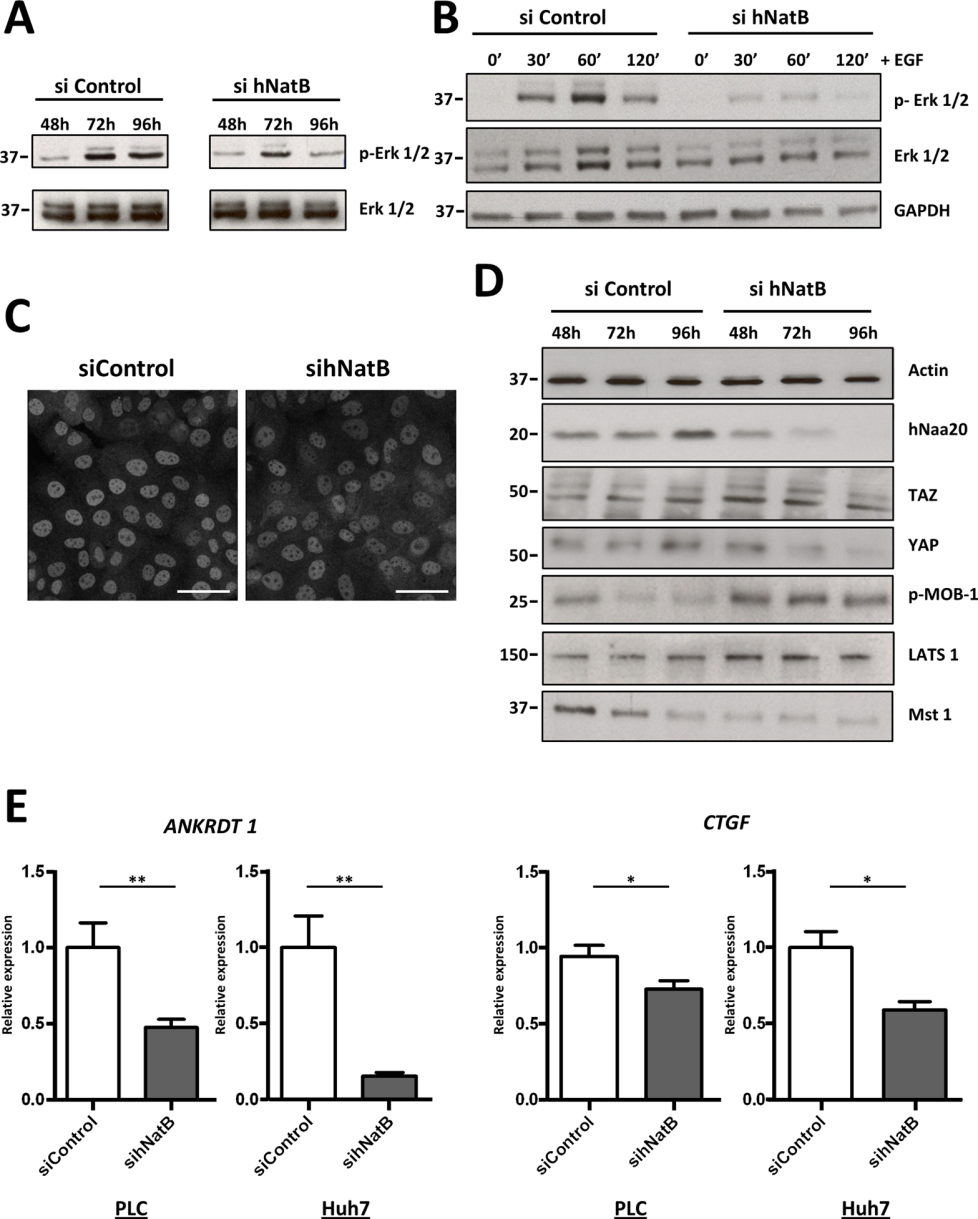
ERK1/2 and Hippo/YAP signaling pathways are inactivated when NatB is inhibited PLC/PRF/5 cell lysates were prepared and ERK1/2 activation was analyzed by western blot 48, 72 and 96 hours after siRNA transfection (**A**) or after treating the cells with EGF (50 ng/ml) for 30, 60 and 120 minutes (**B**). YAP localization was studied by immunofluorescence in PLC/PRF/5 cells transfected with siControl and si*NAA20* and si*NAA25* (sihNatB) siRNAs 96 hours after transfection (**C**). Hippo/YAP pathway activation was studied by western blot in PLC/PRF/5 cell lysates obtained 48, 72 and 96 hours after siRNA transfection (**D**) and analyzing *ANKRDT1* and *CTGF* mRNA expression by RT-PCR 96 hours after siRNA transfection (**E**). The data are presented as the mean ± SME of three experiments. Statistical significance is shown in figure panels and it was set at *p* < 0.05 (*) and *p* < 0.01 (**) value. Unpaired *t*-tests were performed. Scale bar: 25 μm.

The impairment of tumor cell proliferation and cytoskeletal function observed in hNatB-depleted HCC cells led us to investigate critical signaling molecules involved in cellular responses to growth factors and mechanical stimuli, including the ERK1/2 and Hippo/YAP pathways. We found that hNatB knock-down markedly reduced ERK1/2 phosphorylation at 72 h and 96 h after siRNA transfection (Figure 5A) and that EGF-induced ERK1/2 activation was severely blunted in hNatB-deficient cells (Figure 5B, Supplementary Figure 6B). In a multicellular context, mechanical forces are the dominant regulators of the activity of YAP, a highly oncogenic transcription factor expressed in HCC [24]. YAP is transcriptionally active when it is localized in the nucleus, and it is inactive in the cytoplasm. We observed that YAP is localized predominantly in the nucleus in control cells but shuttles to the cytoplasm upon hNatB silencing (Figure 5C). When we examined several of the main components of the Hippo pathway, we observed that hNatB downregulation induced Mob-1 phosphorylation, which is an event associated with YAP inhibition [24] (Figure 5D). To verify whether hNatB depletion reduces YAP transcriptional activity we analyzed two YAP target genes, *ANKRD1* and *CTGF* in PLC/PRF/5 and Huh7 cell lines (Figure 5E) and found that both genes were markedly downregulated, confirming that the YAP pathway is inactivated when hNatB is inhibited. Taken together, these findings indicate that both the ERK1/2 and YAP pathways are abrogated when hNatB is inhibited.

### Tropomyosin dysfunction partially mediates the anti-oncogenic effects of hNatB inhibition

**Figure 6:**
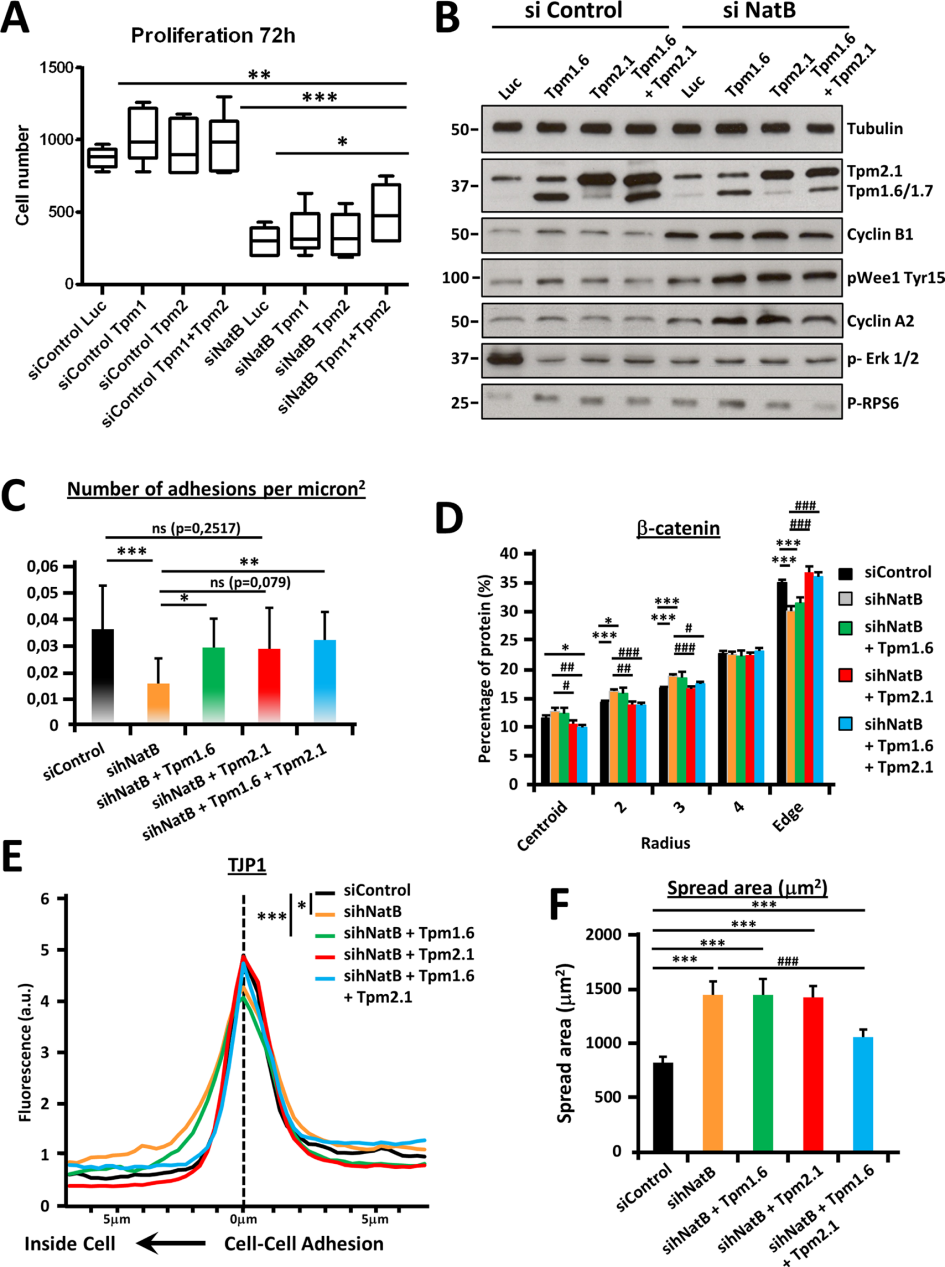
Tropomyosin 2.1 and 1.6 functional deficiencies mediate proliferation and cytoskeletal defects associated with hNatB downregulation PLC/PRF/5 cells transduced with control (siControl) and *NAA20* and *NAA25* (sihNatB) siRNAs were transfected with expression vectors for luciferase (Luc), tropomyosin 1.6 (TPM 1.6), tropomyosin 2.1 (TPM 2.1) or both tropomyosins (TPM 1.6 + TPM 2.1). 72 hours after siRNA transfection cell proliferation was evaluated with Cell Titer-Glo viability Assay (*n* = 3) (**A**) and 24 hours later cell lysates were prepared and cell cycle regulating proteins were analyzed by western blot (**B**). In addition, 96 hours after siRNA transfection cells were fixed and stained for immunofluorescence analysis. Focal adhesions (Vinculin), tight junctions (TJP-1) and adherens junctions (β-catenin) were visualized and the number of adhesions per micron^2^ (≥ 13626 mm^2^ *n* ≥ 7) (**C**), percentage of β-catenin located in equal cell concentric areas (*n* ≥ 21) (**D**) and TJP-1 presence at the cell-cell contact point (*n* ≥ 10) (E) were quantified. Cell spread area corresponds to the average cell surface in mm^2^ in all experimental conditions (*n* ≥ 29) (**E**). The data are presented as the mean ± SME. Statistical significance is shown in figure panels and it was set at *p* < 0.05 (*), *p* < 0.01 (**) and *p* < 0.001 (***) value.

Previous studies have shown that one of the main consequences of NatB knockdown in yeast, flies and mammalian cells is functional impairment of tropomyosin due to absent N-terminal acetylation [3, 25, 26]. We then investigated the implication of TPM 2.1 and TPM 1.6 in the disruption of the cell cycle and the actin cytoskeleton in HCC cells when hNatB expression was suppressed. Cells transduced with control or *NAA20* and *NAA25* siRNAs were transfected with expression vectors expressing luciferase (Luc), tropomyosin 1.6 (TPM 1.6), tropomyosin 2.1 (TPM 2.1) or both tropomyosins (TPM 1.6 + TPM 2.1). After 72 hours, cell proliferation, focal adhesions and cell-cell contacts were evaluated (Supplementary Figure 7). We found that co-overexpression of both TPM 1.6 and TPM 2.1 induced partial recovery of the proliferative activity of hNatB-silenced PLC/PRF/5 cells (Figure 6A). Accordingly, TPM 1.6 and TPM 2.1 co-overexpression was not enough to normalize the expression of cell cycle regulating proteins, such as Cyclin B1 and Cyclin A2, or ERK1/2 and WEE phosphorylation, which is deregulated when hNatB is inhibited (Figure 6B). However, TPM 1.6, and to lesser extent TPM 2.1, was sufficient to reconstitute focal adhesions (Figure 6C) and TPM 2.1, but not TPM 1.6, expression restored β-catenin localization, normalizing its presence at the edge of the cell (zone 5) and in the cell cytoplasm (zones 2 and 3) (Figure 6D). Moreover, TPM 2.1 reconstitutes TJP1 distribution at the cell-cell junction, indicating that adherens junctions and tight junctions share a role for TPM 2.1 in maintaining their structure (Figure 6E). Additionally, co-overexpression of both tropomyosins, TPM2.1 and TPM1.6, is required to reverse the effects of hNatB inhibition on cell size and mTOR activation, linking cell proliferation and size (Figure 6B, 6F).

TPM 1.6 and TPM 2.1 N-terminal acetylation is required for maintaining focal adhesions, tight and adherens junctions structure, proper cell size and cell proliferation in HCC cells.

## DISCUSSION

N-terminal acetylation is a co/post-translational modification of proteins that is essential for their stability and function. hNatB mediates N-terminal acetylation of specific proteins, which are recognized by specific amino acid sequence at the N-terminus. There is an increasing number of proteins that depend on their N-terminal acetyl group for proper functioning [27], including NatB substrates [28, 29]. Here, we show that hNatB is upregulated in a substantial proportion of HCC tumors and that this occurs in association with microscopic vascular invasion. Microscopic vascular invasion is a morphological feature linked to tumor progression and poor prognosis [30], and it is characterized by the presence of tumor emboli in a portal radicle vein, large capsule vessels or in a vascular spaces lined with endothelial cells [31]. Interestingly, both subunits were up- or downregulated in 74% of analyzed tumor samples, indicating the expression of both subunits is co-regulated probably as a consequence of NAA20 degradation when it is not interacting with the accessory subunit NAA25 [13]. *In vitro* studies aimed at analyzing whether an hNatB blockade could exert anti-oncogenic effects on HCC showed that hNatB depletion reduces N-terminal acetylation on proteins [3] and it caused marked cytoskeletal dysfunction in HCC cells accompanied by impaired proliferation and cell cycle progression. Upregulation of NatB expression in HCC can increase tumor cell aggressiveness facilitating microvascular invasion as consequence of an increased proliferation rate or invasion potential.

Consistently, it was found that suppression of hNatB expression in human hepatocellular carcinoma PLC/PRF/5 cells caused profound reduction in the levels of both tropomyosin isoforms and CDK2, two hNatB substrates that are critical regulators of cytoskeletal function and cell cycle progression, respectively. However, their gene expression is either not affected (*CDK2*, *TPM 2.1*) or increased (*TPM 1.6/1.7*) indicating that their stability is impaired when they are not acetylated on the N-terminal residues. Accordingly, previous reports have suggested that protein stability comes from the N-terminus, which is governed by the N-end rule pathway that associates the half-life of a protein to the identity of its N-terminal residue [32].

Abundant evidence in a variety of cell types from yeast to humans, shows that tropomyosin N-terminal acetylation is essential for a functional actin cytoskeleton [3, 25, 26, 33, 34]. It has been shown that N-terminal acetylation of tropomyosin stabilizes the coiled-coil formation in the first 30 amino acids, which is essential for tropomyosin to assemble around the actin filaments [35]. Accordingly, the fusion of a peptide to the N-terminal end of TPM 2.1 disrupts dimer formation, and this prevents TPM2.1 from binding to F-actin, which results in cytoskeleton disorganization and delayed cytokinesis [36]. In agreement with these findings, a genetic linkage between the NatB subunits and tropomyosin in yeast and flies also suggests a functional interaction between these proteins [34, 37]. Several studies demonstrated that tropomyosin has essential roles in focal adhesions and stabilization of cell-cell junctions [16]. We have shown that two tropomyosin isoforms, TPM 1.6 and TPM 2.1, depend on N-terminal acetylation to be stable and functional and are important for focal adhesion formation in hepatoma cells whereas TPM 2.1 is involved in maintaining adherens and tight junction structure, retaining TJP1 and β-catenin at the cell-cell junctions. Indeed it has been reported that TPM 2.1 is part of focal adhesion structure, while TPM 1.6/1.7 integrates with actin stress fibers that generate focal adhesions [17].

The actin cytoskeleton is implicated in the control of numerous signaling pathways that govern cell proliferation [38]. Two pathways connecting mechanical forces and cell growth are the Ras-MAPK-ERK and Hippo pathways, and these require functional actin filaments to control cell proliferation [24, 39]. Importantly, we found that hNatB silencing abrogated both ERK1/2 phosphorylation in non-stimulated HCC cells and ERK1/2 activation in EGF-stimulated cells. Along with ERK inactivation, hNatB inhibition resulted in degradation of the oncogene YAP degradation and downregulation of YAP target genes. These effects were accompanied by impeded formation of actin fibers and a consequential blockade of cytoskeleton-associated functions, such as cellular motility, cellular interaction with the extracellular matrix, cell-cell contacts and cell proliferation.

Several reports have shown an inverse correlation between cell proliferation and cell size [40], and inhibition of hNatB expression increases cell size and reduces proliferation. These two effects are reversed when both tropomyosins are co-overexpressed, revealing a link between NatB expression, tropomyosin function, cell size and proliferation. mTOR and its downstream substrate RPS6 have been demonstrated to control cell size and proliferation [20]. In MEFS, there is an inverse correlation between cell proliferation and RPS6 phosphorylation [41], and in Xenopus eggs, it was found that Ras-induced cell cycle arrest is associated with increased RPS6 phosphorylation [42]. We observed that upon hNatB inhibition, the mTOR pathway is activated by increased RPS6 phosphorylation [20]. Interestingly, RPS6 phosphorylation decreases when both tropomyosins are overexpressed supporting the concept that tropomyosins mediate the effects of the mTOR pathway on cell size and cell proliferation.

Our study shows that NatB activity is necessary for human hepatoma cell proliferation and tumor formation when HCC cells are transplanted subcutaneously in mice. In addition to disrupting the actin cytoskeleton, NatB ablation impinges on cell cycle dynamics by limiting DNA synthesis and progression through the S and G2/M phases. hNatB depletion prevents CDK2 N-terminal acetylation, making this protein less stable and reducing its cellular levels. Inhibition of CDK2 activity arrests cells in late the S and G2 phases in association with increased CDC2 Y15 phosphorylation and inactive Cyclin B/CDC2 [22, 43]. Degradation of CDK2 that is not N-terminal acetylated is associated with active WEE1 stabilization and CDC2 inactivation due to blockage of the G2/M transition. Moreover, the timing of Cyclin B/CDC2 activation, which is essential for driving mitotic events, has been shown to be dependent on Cyclin A/CDK2 activity [44], which is reduced upon hNatB inhibition.

The actin cytoskeleton is an attractive target for anti-cancer chemotherapeutic strategies, but interference with actin polymerization was found to be associated with intolerable cardiac and respiratory muscle toxicity [15]. However, it seems possible that alternative strategies directed to alter the function of cytoskeletal regulatory proteins might display significant antioncogenic activities with acceptable side effects. Here, we have shown that impairing tropomyosin function by implementing a NatB blockade in tumor cells makes them to stop growing and lose tumorigenicity. Interestingly, high molecular weight tropomyosin isoforms are downregulated in many cell transformation events in association with actin cytoskeleton reorganization [45], including HCC development [46]. Changes in actin cytoskeleton properties in tumor cells, such as reducing the expression of high molecular weight tropomyosin isoforms, can make these cells more sensitive to modifications that reduce tropomyosin function. There is increasing evidence of physical interactions between actin cytoskeleton components and the signal transduction pathways that regulate their function. One tropomyosin isoform, TPM 3.1, is involved in regulation of the MAPK/ERK signaling pathway, helping pERK nuclear translocation and governing cell proliferation [47]. We have observed that downregulation of NatB expression in an HCC cell line reduces TPM 1.6/1.7 and TPM 2.1 levels, which affects mTOR signaling but not ERK phosphorylation. There are diverse actin filament populations with distinct functions partially controlled by actin filaments of tropomyosin isoforms [17]. We provide new evidence that supports the regulatory role of distinct signal transduction pathways in tropomyosin isoforms to define different actin filament population functions.

Our data revealed that NatB inhibition affects N-terminal acetylation and function of TPM 1.6/1.7, TPM 2.1 and CDK2, leading to dysfunction of the actin cytoskeleton, impairment of cell cycle progression and abolition of tumorigenicity. Thus, the hNatB blockade represents a new avenue deserving consideration for the treatment of advanced hepatocellular carcinoma.

## MATERIALS AND METHODS

### Human liver samples

27 paired samples of HCC and NT tissue were obtained from patients who underwent curative surgical resection and donated samples to the University of Navarra Biobank. Tumors were classified as Barcelona Clinic Liver Cancer (BCLC) stage A (74%) and B (26%). All patients provided written informed consents, the study protocol conformed to the ethical guidelines of the 1975 Declaration of Helsinki was approved by institutional review board of University of Navarra (065-2012) and samples were provided by the University of Navarra Biobank (BB-13-01). Informed consent was obtained from each patient and the study protocol conformed to the ethical guidelines of the 1975 Declaration of Helsinki as reflected in a priori approval by the appropriate institutional review committee.

### Antibodies

Antibodies recognizing cofilin (FL166), p-cofilin (H2), p53 (DO-1) and YAP (63.7) were obtained from Santa Cruz Biotechnology (Santa Cruz, CA, USA). Antibodies recognizing β-catenin (D10A8), ciclin A2 (BF683), ciclin B1 (D5C10), ciclin D1 (92G2), p-Cdc2 (10A11), CDK2 (78B2), E-cadherin (24E10), p-Erk 1/2 (197G2), YAP (D24E4), LATS1 (C66B5), P-MOB1 (D2F10), p21 Waf1/Cip1 (12D1), p-Rb (S807/811) (#9308), RhoA (67B9) and p-Wee (Ser642) (D47G5 )were obtained from Cell Signaling. Erk 1/2 (06-182) antibody was from Millipore. Anti-β-actin (A2228) and anti-vinculin (V9131) antibodies were supplied from Sigma-Aldrich (St Louis, MO, USA) and anti-ZO-1 (33-9100) and anti-Occludin (33-1500) antibodies were obtained from Invitrogen. An antibody directed against GAPDH (MCA2427) was provided from AbD serotec (BioRad). The antibody specifically recognizing Naa20 (158071AP) was purchased from Protein Tech and an antibody against Naa25 (HPA039322) was purchased from SIGMA. Anti-α-tubulin (T6199) and anti-tropomyosin (T2780) were obtained from SIGMA. The secondary antibodies used were anti-rabbit IgG HRPO (Cell Signaling) and an anti-mouse IgG HRPO (SIGMA).

### Cell culture and siRNA transfection

Huh7 (HB-8065), PLC/PRF/5 (CRL-8024) and SK-Hep1 (HTB-52) were cultured in DMEM supplemented with 10% (vol/vol) dialyzed FBS (Invitrogen), 100 units/mL penicillin (Invitrogen), and 100 μg/mL streptomycin (Invitrogen) at 37°C and in 5% CO2.

Scrambled control siRNA or siRNAs specifically directed against Naa20 and Naa25 were purchased from Invitrogen. The siRNAs transfection was carried out using RNAiMAX Lipofectamine (Invitrogen) in accordance with the manufacturer's procedures. Transfection complexes were added to the cells at a final oligonucleotide concentration of 20 nM.

To disturb actin microfilaments, PLC and Huh7 cells were grown in the presence of cytochalasin B 0.2 μM (Sigma-Aldrich) for the indicated periods of time changing the medium every 24 h. The drugs’ diluent (DMSO) was used as a negative control.

### Immunofluorescence

For staining cytoskeletal proteins cells were fixed with 4% (wt/vol) paraformaldehyde for 15 minutes, permeabilized with 0.1% Triton X-100 and incubated with the following primary antibodies: anti–α-catenin (23B2, Cell Signaling), anti- β-catenin (D10A8, Cell Signaling), anti-paxillin (sc-5574, Santa Cruz), anti-occludin (33-1500, Invitrogen), anti-vinculin (V9131, SIGMA), anti-YAP (D24E4, Cell Signaling) and anti-ZO-1 (33-9100, Invitrogen), followed by incubation with anti-mouse IgG Cy3 conjugated developed in sheep (Sigma) or anti-rabbit IgG Alexa488 conjugate developed in donkey (Molecular Probes). All incubations were performed for 30 min at 37°C. To stain actin stress fibers, cells were incubated with Oregon Green 448 phalloidin (Invitrogen) for 15 min at 20°C. Stained cells for each preparation were mounted with Vectashield Mounting Medium with DAPI (Vector Laboratories). Samples were analyzed using an Axiovert 200M confocal LSM 510 META Zeiss microscope with a 40× objective.

### Western blot analysis

Whole-cell lysates were prepared according to the established procedures. Proteins were resolved by sodium dodecyl sulfate–polyacrylamide gel electrophoresis, immunoblotted with the antibody of interest and visualized using an ECL chemiluminescence detection kit (Amersham Biosciences, Amersham, UK). Obtained western-blot bands were quantified using Image Studio Lite software. At least three independent experiments were carried out with separate samples.

### Real-time PCR assays

Total RNAs were extracted with the Trizol (Invitrogen, Carlsbad, CA, USA). Reverse transcription was performed as previously reported [41]. Real-time polymerase chain reactions were performed with iQ SYBR Green supermix (Bio-Rad) in a CFX96 Real-Time System (BioRad), using specific primers for each gene: *CDK2* F 5′GACACGCTGCTGGATGTCA, R 5′CGGAAGAGCTGGTCAATCTCA; *ANKRD1* F 5′AG TAGAGGAACTGGTCACTGG, R 5′TGGGCTAGAAG TGTCTTCAGAT; *CTGF* F 5′AGGAGTGGGTGTGTGA CGA, R 5′CCAGGCAGTTGGCTCTAATC; *GAPDH* F 5´ACCACAGTCCATGCCATCAC, R 5′TCCACCAC CCTGTTGCTGTA; *TPM2.1* F 5´GACGTCCCAGTC CCGCTCCG, R 5′CTCCTGGGCCTCCTTCACGG; *TPM1.6* F 5′TGGAGCTGGCAGAGAAAAAG, R 5′TTC AAGCTCGGCACATTTGC; *AXIN2* F 5′AGTGCAA ACTTTCGCCAACC, R 5′GGTTCTCGGGAAATGA GGTA and *CCND1* F 5′CCGCCGAGGAGGAGGAAGA, R 5′CCACCGCTCAGGGTTATGCA. The amount of each transcript was quantified by the formula: 2^ct(β-GAPDH)−ct(gene)^, with ct being the point at which the fluorescence rises appreciably above the background fluorescence.

### Cell proliferation assay

To determine the rate of cell proliferation, Cell Titer-Glo Luminescent Cell Viability Assay Titer-GLO kit (Promega) was used. Cells were seeded in 96-well plate and transfected with different siRNAs according to the protocol described in the previous section. For measurement, 50 μl of medium from each well were removed and added to 50 μl of CellTiter-Glo proliferation reagent. Then, the plate was shaken vigorously on a shaker plate for 2 minutes and absorbance of the product formed was measured on a luminescence microplate reader (Orion, Berthold Detection system).

### Analysis of cell cycle distribution

The proliferative capacity of the cells was determined by flow cytometry assay using Click-iT EdU kit (Invitrogen™/Molecular Probes) following the manufacturer's instructions. Cells were incubated with the modified thymidine analogue EdU (5-ethynyl-2′-deoxyuridine) in the culture medium at a concentration of 10 μM to visualize DNA synthesis for 2 hours at 37°C and 5% of CO2 in the cell incubator. Incorporated Edu was detected with Alexa Fluor 647 azide according to the staining protocol provided by Invitrogen. The samples were analyzed with BD FACSCanto™ cytometer (BD Biosciences) using an excitation wavelength of 633 nm and an emission filter of 660 nm. Data were analyzed with software version 7.6.3 FlowJo.

For synchronization of cells in the early S phase double thymidine block was used. To do this, cells were incubated for 14 hours with thymidine 2 mM in the culture medium. Then, thymidine medium was removed and fresh medium with 24 mM deoxycytidine (Sigma) was added and incubated for 9 hours. Thymidine blockade and deoxycytidine incubation were repeated once more as described above. Then, cells were washed with phosphate buffered saline (Gibco) and cell cycle flow cytometry analysis was conducted under the same conditions described above, including an additional incubation for 1 hour in fresh medium before adding EdU to avoid deoxycytidine interference.

### Xenograft tumor generation

Animal studies were conducted in accordance with the institutional guidelines for care and use of laboratory animals and were approved by the competent authority (018–11). All animals received humane care according to the criteria outlined in the “Guide for the Care and Use of Laboratory Animals” prepared by the National Academy of Sciences and published by the National Institutes of Health (NIH publication 86–23 revised 1985). Animal experiments were performed conform to the Animal Research: Reporting of *In Vivo* Experiments (ARRIVE) guidelines. To generate xenograft tumors, 5 × 10^5^ siControl or siNatB transfected Huh7/PLC/SK-Hep1 cells were subcutaneously injected into the right or left of 6–8 week old BALB/c Rag2^tm1Fwa^ Il2rg^tm1Amk^ /J male mice (6–7 animals) flank respectively, 48 hours after siRNA transfection or Cytochalasin B (50 μg/ml) treatment. Tumor volumes were calculated using the formula: tumor volume (cm^3^) = 0.52 × (width) 2 × (length).

### Morphometric analysis and quantification of focal and cell-cell adhesions

Cell spread areas, the percentage of beta catenin intensity per section and vinculin focal adhesion properties were quantified using previously described [42] custom-made code written in Matlab (The Mathworks Inc., Natick, MA, USA).

The percentage of total beta-catenin intensity per section was defined as the sum intensity of all pixels contained in a section divided by the sum intensity of beta-catenin across the whole cell. Briefly, to quantify intensity independently of cell size or shape, cell morphology was first transformed into a uniform circular distribution where the center and the perimeter correspond to the cell centroid and perimeter, respectively. This circular distribution was then divided into five ring-shaped sections of equal area from the center to the edge. The resulting sum of each section was divided by the sum of the intensity across the entire cell [48].

To measure the disruption of tight junctions, a custom-made code was written in Matlab to measure the local mean ZO-1 intensity across cell-cell adhesions of each cell. First, cell-cell adhesions corresponding to one cell and the area close to these adhesions were segmented. Then, the mean intensity was then quantified as a function of the distance to the adhesion. Vinculin focal adhesions were segmented and then their area was quantified following the same procedure as described previously [42].

### Statistical analysis

Statistical significance was assessed using Prism 5 (GraphPad Software) and IBM SPSS Statistics. When the number of samples of each variable was under 10 it was conducted a statistical test for non-parametric Student t followed by a U Mann- Whitney test to compare two groups. When the number of samples analyzed was greater than 10, the statistical significance of the differences between groups was determined from the mean and the SD using unpaired, two-tailed *t* test. ZO-1 distribution statistical significance between the control and experimental groups was determined using Shapiro-Wik normality test, equal variance test and a paired *t-test*. The number of cells examined for β-catenin distribution was ≥ 21, for ZO-1 distribution *n* ≥ 10, for spread area *n* ≥ 29 and for focal adhesion analysis ≥ 13626 mm^2^ and *N* ≥ 7. Statistical significance was set at *p* < 0.05 (*), *p* < 0.01 (**) and *p* < 0.001 (***) value.

## SUPPLEMENTARY MATERIALS FIGURES



## Abbreviations

HCC: hepatocellular carcinoma
KAT: lysine acetyltransferase
NAT: alpha-aminoterminal acetyltransferase
TPM 1.6: tropomyosin 1.6
TPM 2.1: tropomyosin 2.1
BCLC: Barcelona Clinic Liver Cancer
RT-PCR: real-time reverse-transcriptase polymerase chain reaction
RPS6: Ribosomal Protein S6
