# Supplementary material for: NatB-mediated protein N-α-terminal acetylation is a potential therapeutic target in hepatocellular carcinoma
Source: Oncotarget. 2017 Apr 21;8(25):40967–81. doi: 10.18632/oncotarget.17332 (PMC5522283; doi:10.18632/oncotarget.17332)
Supplement: Supplementary file 1 [file oncotarget-08-40967-s001.pdf]

NatB-mediated protein N-α-terminal acetylation is a potential therapeutic target in hepatocellular carcinoma

Supplementary Materials

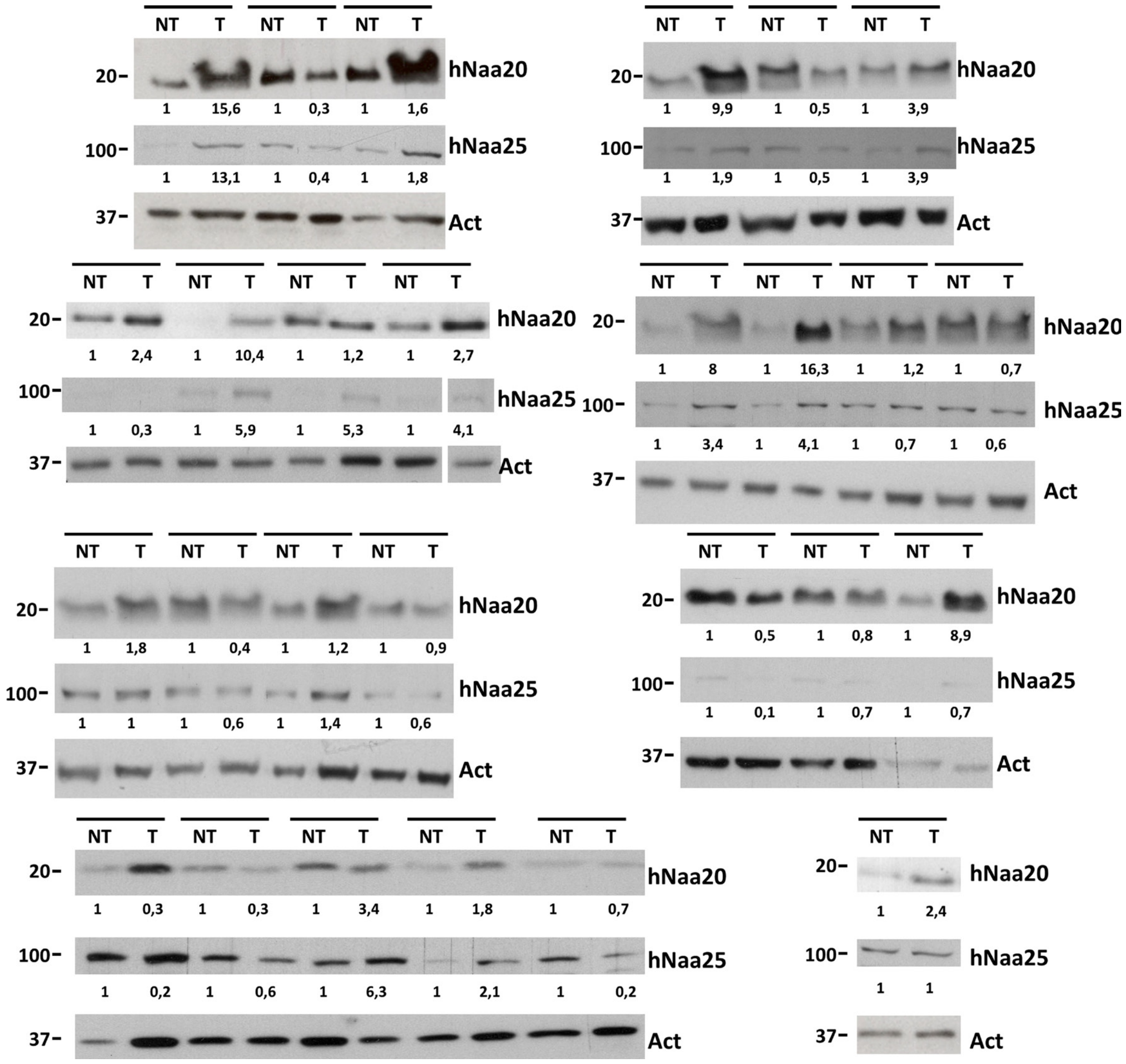

Supplementary Figure 1: hNatB protein expression in human hepatocellular carcinoma (HCC) and non-tumor liver tissue. Western blot of hNatB subunits, NAA20 and NAA25, in the non-tumor (NT) and tumor (T) liver tissue of patients with HCC. Images were quantified, normalized with obtained actin values and compared NT vs T paired samples, being considered NT sample as 1.

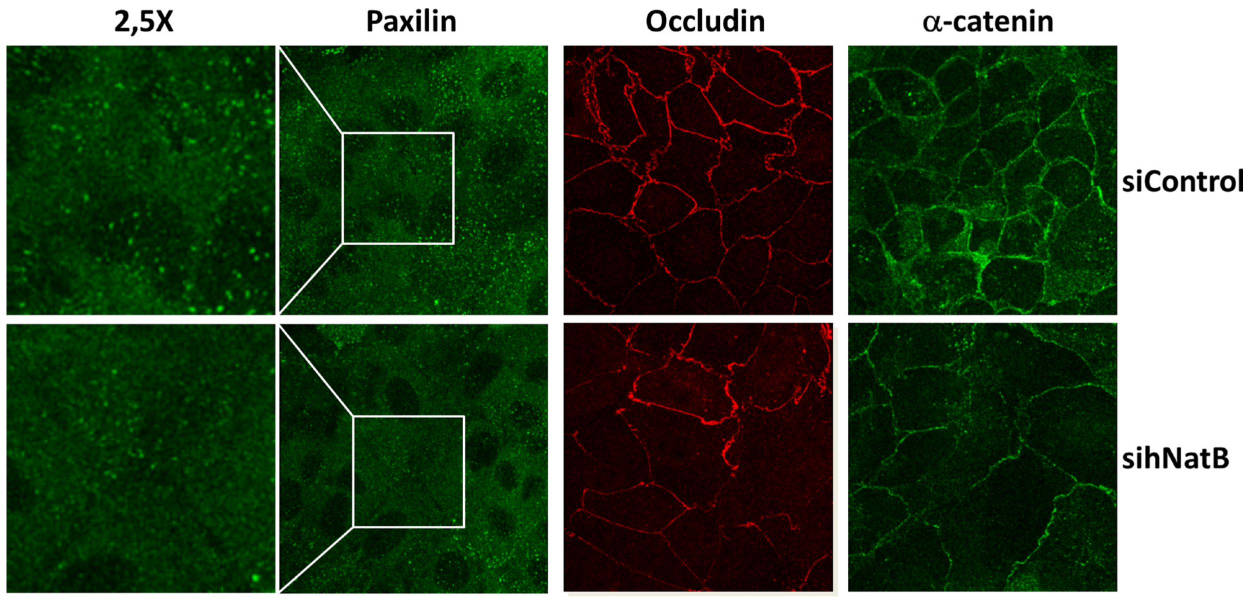

Supplementary Figure 2: hNatB regulates focal adhesion and cell-cell contact structures architecture. PLC/PRF/5 cells were transfected with negative control siRNA (siControl) or *NAA20* and *NAA25* siRNAs (sihNatB) and 96 hours later were fixed with paraformaheldyde and analyzed by immunofluorescence with specific antibodies for focal adhesions (Paxilin), tight junctions (Occludin) and adherens junctions (α-catenin). All images were obtained with a 63 × objective. Detailed focal adhesions are shown in amplified (2, 5 ×) images.

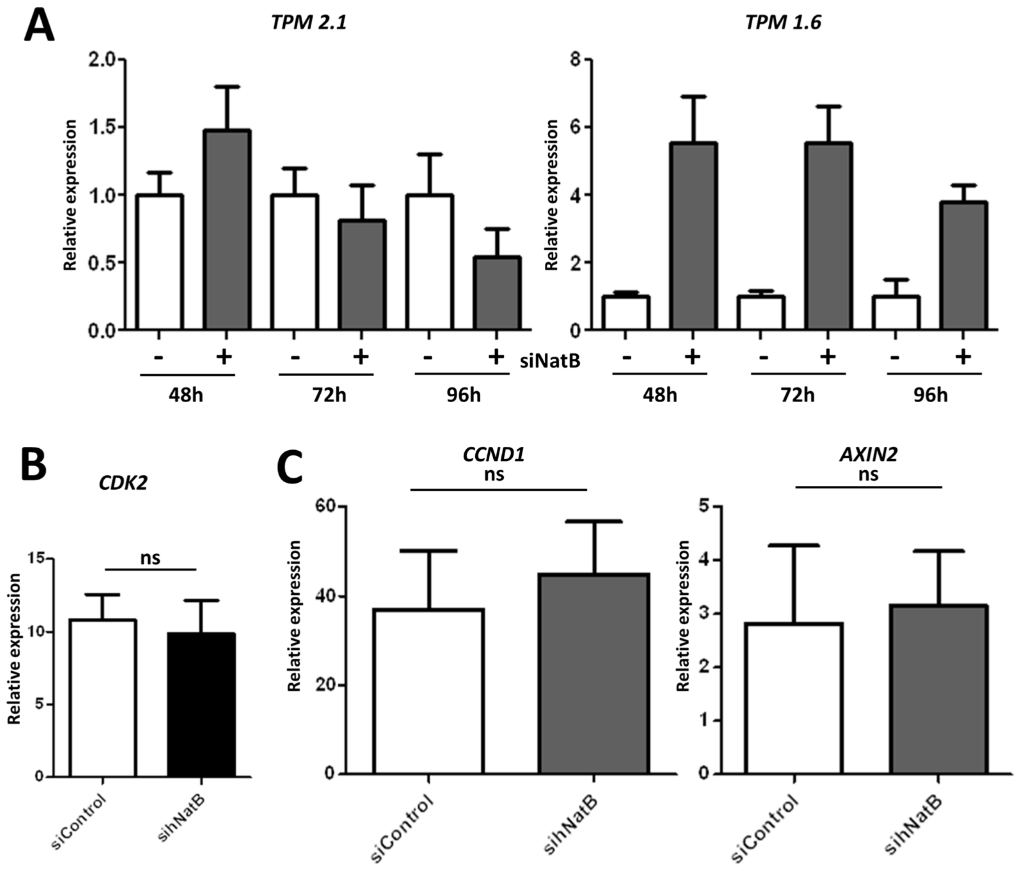

Supplementary Figure 3: hNatB downregulation effects on tropomyosin and CDK2 expression, β-catenin activity and mTOR pathway. hNatB expression was blocked after specific *Naa20* and *Naa25* siRNAs transfection (sihNatB) in PLC/PRF/5 and tropomyosin 2.1 and tropomyosin 1.6 mRNA expression was quantified 48, 72 and 96 hours after transfection (**A**). CDK2 mRNA expression was quantified in PLC/PRF/5 cells 96 hours after control siRNA (siControl) or *NAA20* and *NAA25* siRNAs (sihNatB) transfection (**B**). In addition β-catenin activity was evaluated in PLC/PRF/5 cells 96 hours after siRNAs transfection measuring *CYCLIN D1* (*CCND1*) and *AXIN2* mRNA expression by quantitative RT-PCR (**C**).

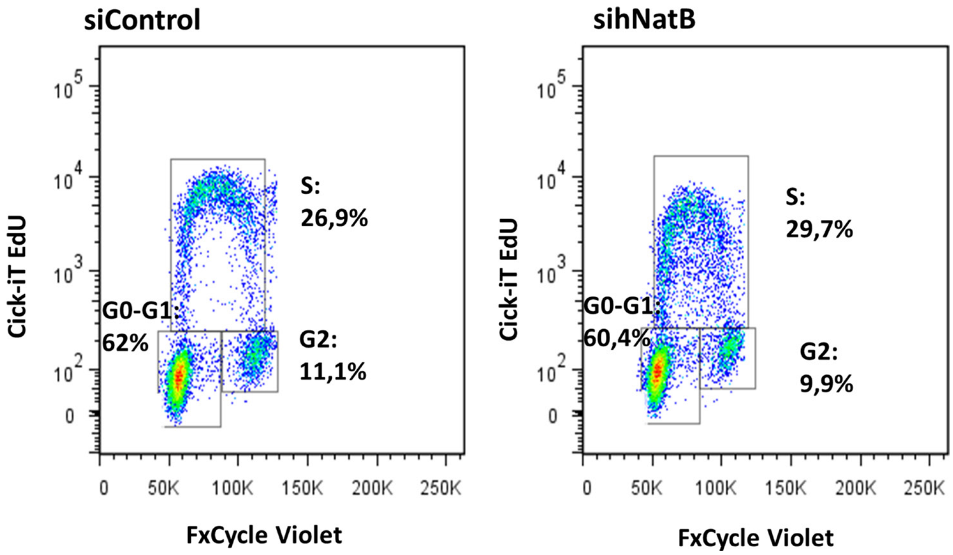

Supplementary Figure 4: Cell cycle phase analysis in PLC/PRF/5 cells after hNatB expression inhibition. 96 hours after control (siControl) and *NAA20* and *NAA25* (sihNatB) siRNAs transfection PLC/PRF/5 cells were incubated for one hour with EdU, harvested and DNA synthesis and cell cycle phases were determined by FACS analysis. Click-iT EdU indicates DNA synthesis and FxCycle Violet stains total DNA.

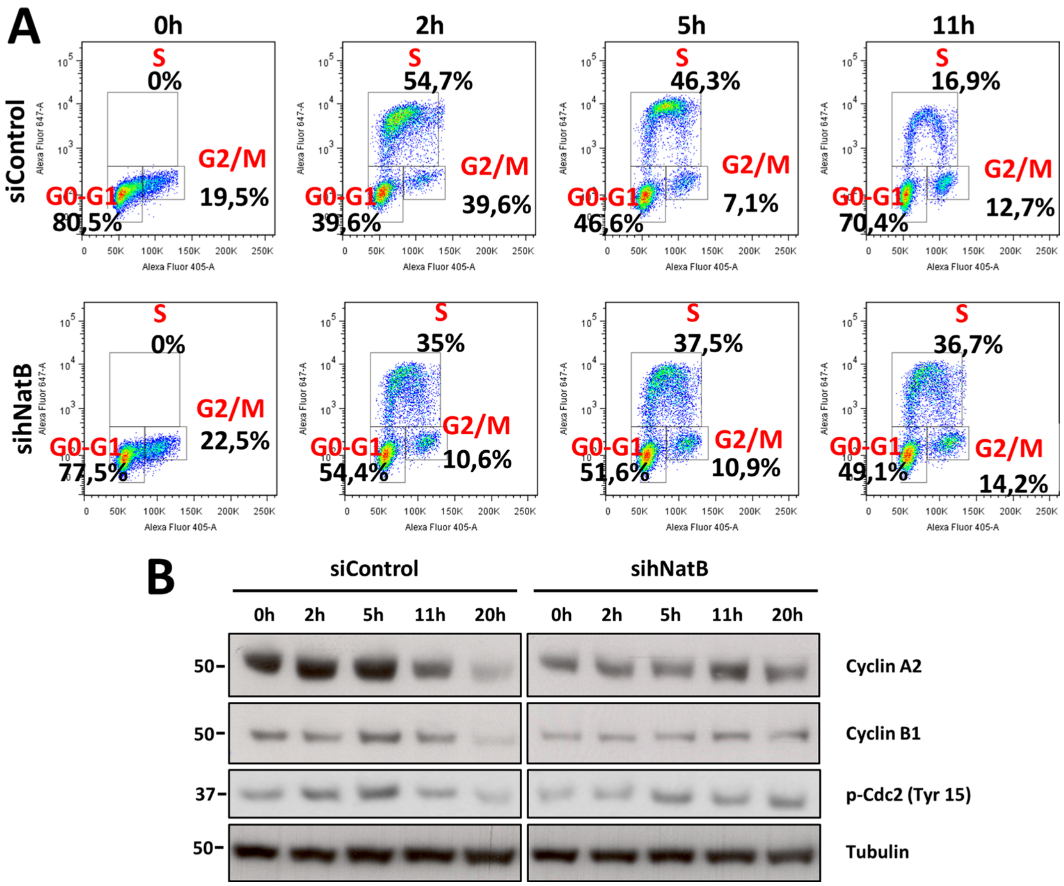

Supplementary Figure 5: Cell cycle phase progression analysis in synchronized PLC/PRF/5 cells after hNatB expression inhibition. siRNA transfected PLC/PRF/5 cells were synchronized in the early S phase with doble thymidine treatment, wash and analyze them by FACSand western blot 0, 2, 5 and 11 hours after cell cycle blockade release. Before cells collection and FACS analysis, they were incubated for 1 hour with EdU for DNA synthesis quantification. Alexa Fluor 647-A represents EdU incorporation and Alexa Fluor 405-A indicates FxCycle Violet total DNA staining (**A**). Cell lysates were prepared and Cyclin A2, Cyclin B1 and CDC2 phosphorylated in tyrosine 15 (p-CDC2 Tyr 15) expression was analyzed at the different time points (**B**).

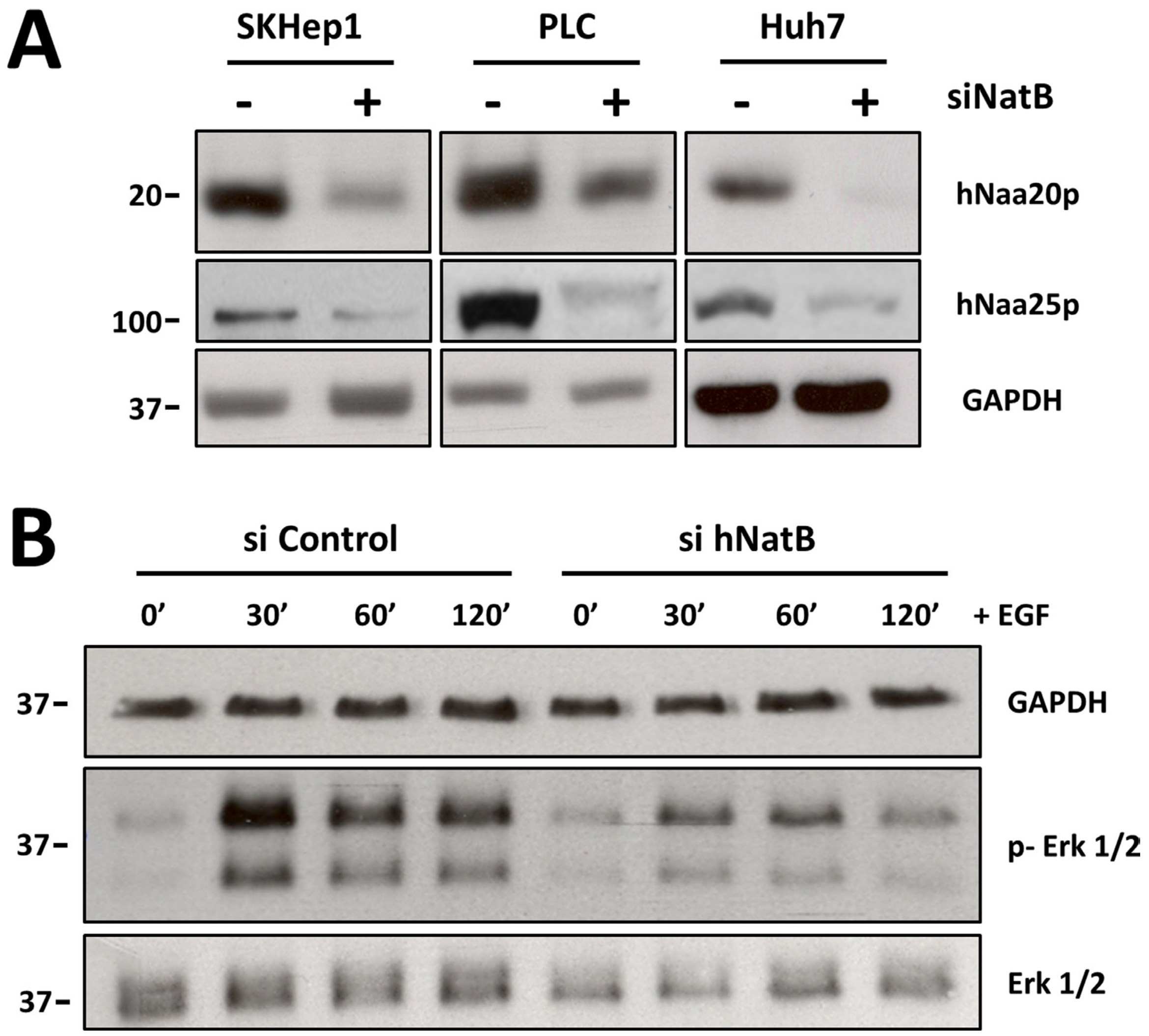

Supplementary Figure 6: Expression analysis of hNatB subunits in diferent cell lines when NatB is inhibited and ERK activation in Huh7 cells. PLC/PRF/5, Huh7 and SkHep1 cell lysates were prepared 96 hours after siRNA transfection and downregulation of hNatB subunits, NAA20 and NAA25, was confirmed by western blot (**A**). ERK1/2 activation was analyzed by western blot 96 hours after control and hNatB siRNA transfection and treating the cells with EGF (50 ng/ml) for 30, 60 and 120 minutes (**B**).

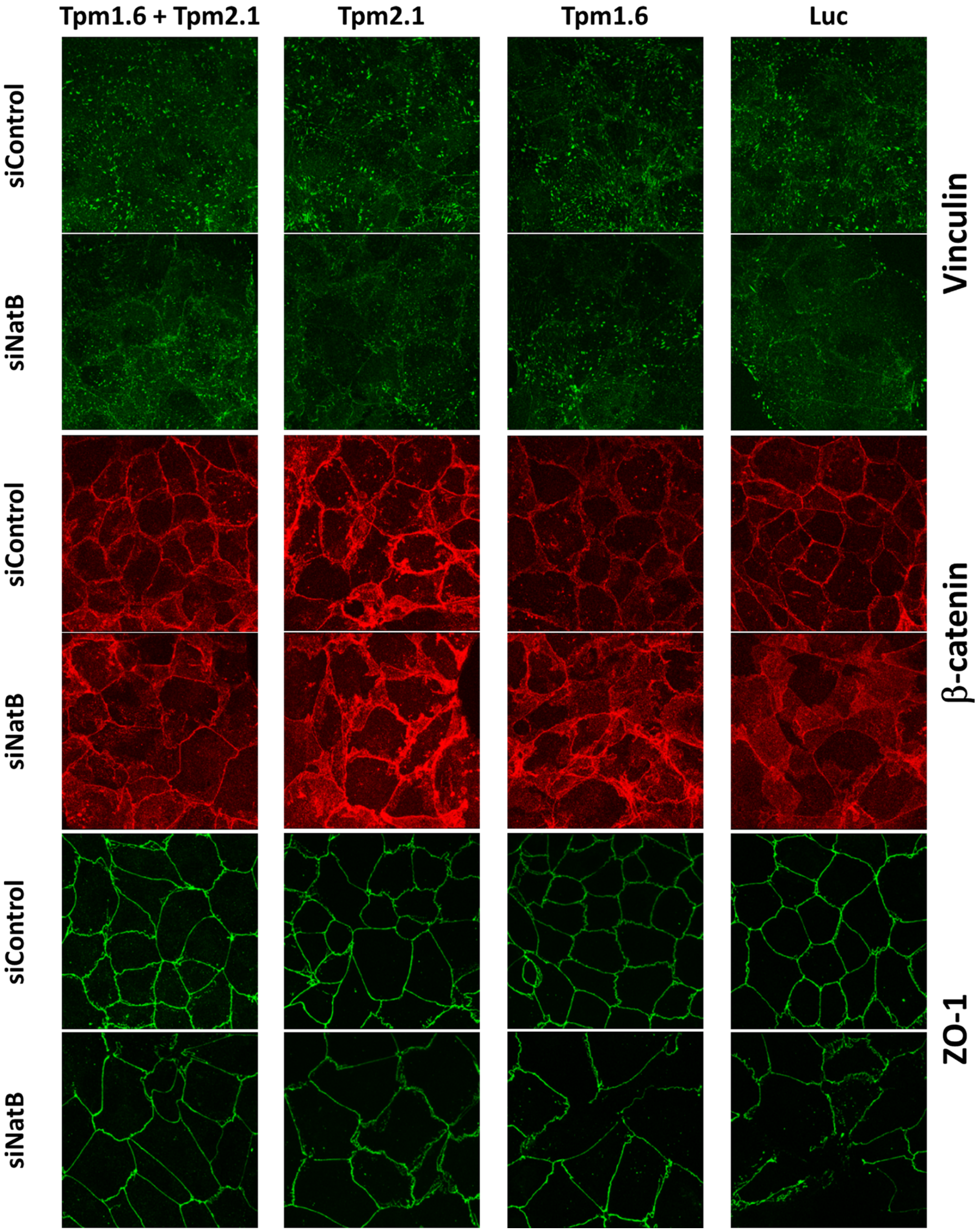

Supplementary Figure 7: Visualization of focal adhesions, tight junctions and adherens junctions when Tropomyosin 2.1 and 1.6 are overexpressed in PLC/PRF/5 cells. PLC/PRF/5 cells transduced with control (siControl) and Naa20 and Naa25 (sihNatB) siRNAs were transfected with expression vectors for luciferase (Luc), tropomyosin 1.6 (TPM 1.6), tropomyosin 2.1 (TPM 2.1) or both tropomyosins (TPM 1.6 + TPM 2.1). 96 hours after siRNA transfection cells were fixed and stained for immunofluorescence analysis. Focal adhesions (Vinculin), tight junctions (ZO-1 or TJP1) and adherens junctions (β-catenin) were visualized. Images were acquired with a 63 × objective.
